# Tachycardia-Induced Cardiomyopathy in a 1-Month-Old Infant

**DOI:** 10.1155/2012/513690

**Published:** 2012-12-18

**Authors:** Joseph C. Mares, Yaniv Bar-Cohen

**Affiliations:** Division of Cardiology, Department of Pediatrics, Children's Hospital Los Angeles, USC Keck School of Medicine, 4650 Sunset Boulevard, Mail Stop No. 34, Los Angeles, CA 90027, USA

## Abstract

Supraventricular tachycardia (SVT) is the most common arrhythmia in children and is especially common in infants. SVT is typically thought of as an acute condition; however, if unrecognized, a persistent tachyarrhythmia can progress to a state of cardiac contractile dysfunction known as tachycardia-induced cardiomyopathy. A high index of suspicion for an underlying arrhythmia is needed in the workup of any patient with new onset heart failure, and the 12-lead electrocardiogram can aid in the diagnosis. While this may be a rare cause of dilated cardiomyopathy and heart failure in children, the condition is usually reversible and should be considered in infants and young children.

## 1. Introduction

Tachycardia-induced cardiomyopathy is a deterioration in ventricular function secondary to sustained tachycardia, which is partially or completely reversible after normalization of the heart rate [1]. Here, we present a case of tachycardia-induced cardiomyopathy due to SVT in a patient with underlying Wolff-Parkinson-White (WPW) whose cardiac systolic function rapidly and completely normalized with control of the tachyarrhythmia. Although our patient did not present in SVT, the rapid improvement in ventricular function in the setting of WPW highly suggested the diagnosis of tachycardia-induced cardiomyopathy, even before SVT was clinically seen.

## 2. Case Report 

A 4-week-old male infant was brought to the emergency room for a 3-day history of non bilious, non bloody emesis with feeds as well as fussiness and tachypnea. The frequency of emesis had increased to after almost every feeding, and he had decreased urine output. He otherwise had no fever, upper respiratory tract symptoms, diarrhea, or sick contacts. He was born at term via uncomplicated vaginal delivery and had previously been well.

On initial physical examination, his weight was 3.9 kg, temperature was 36.5°C, heart rate was 147 bpm, blood pressure was 78/44 mmHg, respiratory rate was 70 breaths/min, and oxygen saturation was 100% on room air. He had mild tachypnea and mild abdominal distention with the liver edge palpated below the costal margin. The remainder of the exam including heart and lungs was within normal limits.

An abdominal ultrasound showed no evidence of pyloric stenosis. Therefore, an oral feeding trial was attempted. Upon reassessment, however, the infant was found to be in moderate respiratory distress. He was placed on oxygen, and a chest X-ray revealed mild cardiomegaly and mild pulmonary edema. A capillary blood gas revealed a pH 6.84, PCO_2_ 64 mmHg, HCO_3_ 11 mEq/L, and base deficit of 24. The infant was intubated, and two normal saline fluid boluses of 20 mL/kg were given. 

An echocardiogram showed a structurally normal heart but with severely decreased right and left ventricular systolic function and a left ventricular shortening fraction of 13% (normal range: 28–44%). The cardiac size was normal with a left ventricular end-diastolic diameter of 2 cm (*Z* score −0.6). Because of the finding of severe ventricular dysfunction, the patient was transferred to the neonatal intensive care unit and started on inotropic support with dopamine and milrinone infusions. 

**Figure 1:**
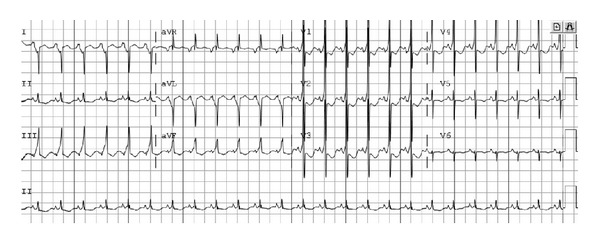
Initial 12-lead electrocardiogram showing sinus rhythm (HR: 150 bpm) and the ECG findings of Wolff-Parkinson-White (WPW) syndrome.

Upon admission, a 12-lead electrocardiogram (ECG) was obtained and showed sinus rhythm with a rate of 150 bpm. A short PR interval of 50 ms was seen as well as a delta wave resulting in a prolonged QRS duration of 110 ms (Figure 1). These findings were consistent with ventricular preexcitation, and a diagnosis of Wolff-Parkinson-White (WPW) syndrome was made. Due to the lack of tachycardia observed at initial presentation, however, a diagnosis of tachycardia-induced cardiomyopathy was suspected but could not be confirmed. Laboratory testing to evaluate inborn errors of metabolism and specific viral serology, and PCR was therefore ordered to possibly delineate an additional underlying diagnosis.

**Figure 2:**
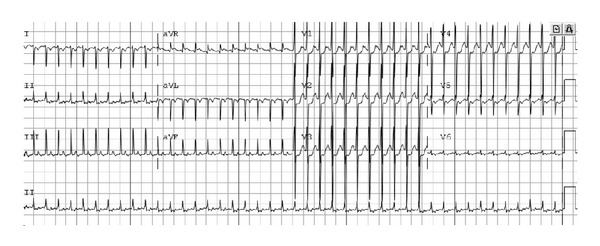
12-lead electrocardiogram obtained during an episode of supraventricular tachycardia (HR: 260 bpm).

A follow-up echocardiogram was done on hospital day (HD) 2 that showed complete normalization of ventricular function with an LV shortening fraction of 44%. The patient was therefore weaned off inotropic support and successfully extubated the next day. Later on HD 3, however, the patient had multiple episodes of supraventricular tachycardia (SVT) to rates of 260 bpm (Figure 2). Vagal maneuvers (application of ice to the face) did not affect the rhythm, and IV adenosine was needed to terminate the SVT. Thereafter, multiple recurrences of sustained SVT occurred over the next 24 hours requiring intervention with ice and/or adenosine.

Oral propranolol was initially started, but, due to persistent episodes of SVT, oral flecainide was initiated on HD 6 (~100 mg/m^2^/day). Except for one very brief self-limited episode of SVT, there were no further sustained tachycardia episodes observed after starting flecainide, and the patient was discharged home on HD 10. He has not had any recurrences on flecainide therapy and has been feeding well without respiratory symptoms. Follow-up echocardiograms have continued to show normal biventricular function. This course confirmed the diagnosis of tachycardia-induced cardiomyopathy due to supraventricular tachycardia in a patient with underlying Wolff-Parkinson-White syndrome.

## 3. Discussion

Tachycardia-induced cardiomyopathy, sometimes referred to as tachycardiomyopathy, is a deterioration in ventricular function secondary to incessant tachycardia. This can lead to dilated cardiomyopathy and symptoms of heart failure if unrecognized. The diagnosis can be made when an improvement in function is seen after normalization of the heart rate [1]. Prevalence and incidence of tachycardia-induced cardiomyopathy are difficult to estimate due to few published case series and because it may be an under-recognized etiology of dilated cardiomyopathy and heart failure in children.

Inciting arrhythmias are usually forms of supraventricular tachycardia including both reentrant and automatic atrial tachyarrhythmias. In young children and infants, the most common etiologies are orthodromic reentrant tachycardia (including patients with WPW), ectopic atrial tachycardia (EAT), and permanent junctional reciprocating tachycardia (PJRT). Atrial fibrillation, atrial flutter, or incessant ventricular tachycardia may also lead to tachycardia-induced cardiomyopathy although these arrhythmias are typically seen in older patients [2].

The typical clinical presentation of tachycardia-induced cardiomyopathy is similar to other forms of heart failure. Infants are recognized on the basis of respiratory distress, diaphoresis, and poor feeding, while older children may present with exercise intolerance, dyspnea on exertion, chest pain, or syncope [3, 4]. Older children may describe palpitations though this is not always reliable. Since the degree of ventricular dysfunction often correlates with the duration and rate of the tachycardia, older children and adolescents typically present with heart failure weeks or months after the onset of tachycardia if the heart rate is only moderately elevated. Infants, however, usually show heart failure symptoms only days after the onset of tachycardia due to the typically much faster ventricular rates during SVT and their inability to verbalize their early symptoms. 

A high index of suspicion for an underlying tachyarrhythmia is needed in the evaluation of a patient with new onset heart failure. Systolic ventricular dysfunction is often the first manifestation of tachycardia-induced cardiomyopathy seen on echocardiogram, followed by left ventricular dilatation after prolonged dysfunction [5]. Also importantly, an electrocardiogram should be obtained on any patient presenting with new onset dilated cardiomyopathy. This may show the usual findings in heart failure of sinus tachycardia, nonspecific ST-T wave changes, and ventricular hypertrophy. While the responsible tachyarrhythmia itself may be observed, other abnormal findings may also be seen even if the child is at a slower rate during the electrocardiogram. Clues to an underlying arrhythmia include an abnormal P-wave axis, frequent atrial or ventricular ectopy, or evidence of Wolff-Parkinson-White syndrome (WPW). 

The mechanisms responsible for the development of cardiomyopathy from incessant tachycardia are unclear, but several theories have been proposed. Abnormal cellular remodeling may occur with sustained tachycardia which diminishes the number of microtubules in the cardiac myocyte and contributes to myocardial contractile dysfunction [6]. Chronic tachycardia may also lead to a depletion of high-energy phosphates and cellular reduction in sarcolemmal sodium/potassium ATPase activity as well as changes in enzyme distribution, may affect calcium handling [7]. Therefore, infants and young children may have more rapid deterioration in function due to lower intracellular calcium reserves. These potential cellular changes can result in ventricular dysfunction, neurohormonal activation, and clinical findings also seen in other etiologies of congestive heart failure in children.

Focusing the differential diagnosis for infants or children presenting in acute heart failure with a structurally normal heart is often challenging because of the multiple possible etiologies. Infectious, familial, metabolic, mitochondrial, toxic, inflammatory, and neuromuscular processes may be responsible although most cases remain idiopathic [3]. Infectious myocarditis is an important consideration in acute cardiomyopathy with viral etiologies, including coxsackie B virus, being the most common offending agents. Genetic and metabolic etiologies such as storage disorders, mitochondrial disorders, and carnitine deficiency should also be considered in infants and young children with new onset cardiomyopathy [8]. Anomalous origin of the left coronary artery from the pulmonary artery (ALCAPA) is another possible diagnosis that can be found by careful echocardiographic examination of the coronary arteries and close evaluation of the ECG for the classic findings of large Q-waves in the lateral leads (I, AVL, V5-6). 

While any patient with new onset heart failure should have tachycardia-mediated cardiomyopathy considered as an underlying etiology, confirming this diagnosis is often challenging. First, as seen with our patient, the responsible tachycardia may not be evident at presentation [2]. In addition, a diagnosis of tachycardia-mediated cardiomyopathy may be difficult to confirm until after normalization or improvement of the ventricular function with control of the tachyarrhythmias [7]. Establishing a cause and effect relationship can be challenging as patients with dilated cardiomyopathy often have elevated heart rates and nearly half may have arrhythmias secondary to the cardiomyopathy [1, 3]. As a result, tachycardia-mediated cardiomyopathy is considered the most frequently unrecognized curable cause of heart failure [7]. In fact, instances have occurred when pediatric patients with “idiopathic dilated cardiomyopathy” were referred for cardiac transplantation then subsequently found to have a tachycardia-induced cardiomyopathy that resolved with treatment of the arrhythmia [9]. 

The main approach to treatment of tachycardia-induced cardiomyopathy is control of the tachyarrhythmia. This may be achieved by the initiation of antiarrhythmic medications in consultation with a pediatric cardiologist since the drug choice depends on the underlying arrhythmia. Often, beta-blocking agents (Class II antiarrhythmic medications by the Vaughan-Williams classification) or digoxin (Class V) are used as first line therapy for preventing recurrence of supraventricular tachycardias [10]. Some persistent arrhythmias may require treatment with stronger antiarrhythmic agents such as flecainide (Class IC) or amiodarone (Class III). Incessant supraventricular tachycardia, however, may be refractory to conventional treatments, and an electrophysiology study with radiofrequency ablation of the arrhythmia substrate may be required for those that do not respond to medical therapy [5]. Time to recovery is generally related to the duration of the tachycardia. With the elimination of the underlying arrhythmia, the recovery of ventricular function is often seen within days to weeks in infants but may take up to 2-3 years in older children that may have had a much longer duration of tachycardia [5]. 

## 4. Conclusions

Tachycardia-induced cardiomyopathy is an underappreciated, but treatable, cause of dilated cardiomyopathy leading to heart failure in pediatric patients with structurally normal hearts. This diagnosis should be considered even when the patient does not present with an arrhythmia since it may not be evident on initial presentation. It is important to consider that although supraventricular tachycardia is generally thought of as an acute medical condition, if unrecognized for an extended duration, it may present with heart failure rather than the symptoms of tachycardia alone. It is therefore essential to obtain an early 12-lead electrocardiogram in the workup of any patient with new onset heart failure. Unlike many other etiologies of heart failure, tachycardia-induced cardiomyopathy may show complete normalization of cardiac function and generally has an excellent prognosis once the arrhythmia is controlled.
